# Innovation capabilities and human development competitiveness in education sector: Evidence from UAE

**DOI:** 10.3389/fpsyg.2022.933432

**Published:** 2022-07-22

**Authors:** Ashraf M. Zedan Al Dulaimi, Sultan Mohamed Al Marzooqi, Asmuliadi Lubis, Norrodzoh Binti Hj Siren, Sayyid Buhar Kassim

**Affiliations:** Department of Dakwah and Human Development, University of Malaya, Kuala Lumpur, Malaysia

**Keywords:** innovation capabilities, higher education, human development index (HDI), UAE, Human Capital Theory

## Abstract

Innovation capabilities and human development in education sector is one of the key focused areas in United Nations Sustainable Development Goals. The current research is novel to integrate and address such constructs in single theoretical framework. Grounded in Human Capital Theory, this study aims to investigate the role of innovation capabilities in human development competitiveness in the education sector of UAE. Based on the qualitative research approach, data were collected through interviews with 70 experts and leaders working in this field of Human Development Index (HDI) and innovation development in the UAE in order to more comprehensively recognize the main dilemmas involved in the phenomenon under investigation. Results indicate a huge potential of growth and improvement in education sector of UAE for supporting country SDG goals. Furthermore, it applied the conceptual statistics of key performance indicators (KPIs) collected to analyze the UAE’s HDI, innovation capabilities, and human development competitiveness from 2014 to 2020. Results revealed insightful lessons for policymakers and scholars working in innovation management and sustainable development goals area. Especially, this research will bring key policy directions and future research avenues for better innovative capabilities development in the education sector in developing and low-income economies.

## Introduction

Education is considered as an important contributor to the development in all developed and developing countries, especially in low-income societies for growth and prosperity. Education is both a source and an outcome of development, and it is crucial to the wider idea of expanding innovation capabilities, which is the pivot of the advancement era. Simultaneously, education is critical to a growing nation’s ability to handle advanced technologies and acquire the potential for inner progress and expansion (Ilechukwu et al., 2014). To put it in another way, education is vital to unlocking a person for social change and long-term human growth. In the twenty-first century at least a worldwide plan of action was described by Deffinika et al. (2021) “Education… should be recognized as a process by which human beings and societies can reach their fullest potential. Education is critical for promoting sustainable development and improving the capacity of the people to address the environment and development issues”. This study is incremental and different from previous studies to shed light on scarcely explored phenomenon, of higher education with innovation capabilities and human development index; how specifically innovation capabilities improve education sectors and reflect in human development indicators (HDI) is the main advance made by this research.

Furthermore, education for skills development affects economic growth and knowledge levels; on the other hand, the use of innovative technology can contribute to creating development opportunities in various innovative programs. Accordingly, the UAE must increase investment in education, especially R&D activities in order to achieve globally outstanding levels through joint cooperation between different sectors (Ali et al., 2010; Farooq et al., 2010; Ain et al., 2016; Abd-Alla et al., 2021). Therefore, the UAE is constantly aspiring to occupy advanced positions at the global level in all areas of human development, including the education sector. In this regard, the UAE was ranked 31st globally in 2020 and made an enormous leap forward between 1990 and 2020, increasing its total HDI from 0.723 to 0.890, with a change rate of 81% (Alfaki, 2018; Conceição, 2020). Moreover, countries seek to achieve these indicators by developing and implementing plans, programs, and various projects, thereby contributing to the application and improvement of international human development standards (Deffinika et al., 2021). This can lead to a country improving its global position ranking, especially in health, education, economics, and well-being, which are the main tools used by people to develop and achieve their aims (Jam et al., 2018; Huo et al., 2019; Qureshi et al., 2020).

Innovation capabilities in itself is a state-of-the-art construct were used in various recent innovation studies. Bridging it together with human capability and development parameters and education sector case makes this research a state-of-the-art contribution related to topic. Moreover, human development has been identified as the true objective and primary engine of sustainability, which aims for carefully tuned financial, societal, and natural consequences, in this study. This acceptance, meanwhile, is not without its difficulties and opposition. Researchers have previously emphasized a company’s resources as a means of innovation, competitiveness, and development. However, the country’s perspective investigations have been rare in literature and this study aims to contribute to better conceptualization. From an available resource perspective without significant funding for education, no country can grow (Jam et al., 2011; Malek et al., 2020; Phale et al., 2021). The strength of a state’s human capital, which is the most significant and vital aspect of creation and processing, is linked to its growth. In addition, contextually discussing the UAE achieved a global innovation ranking of 34th and 1st within the Arab world in 2020 (Dutta et al., 2020). However, the UAE must still overcome certain obstacles and challenges it wish to be a top-10 tier HDI country, such as Norway, Ireland, Switzerland, and Hong Kong. Indeed, the UAE must use both tangible and intangible innovation capabilities to improve and promote its HDI requirements (Alfaki, 2018). Since the UAE’s establishment on December 2, 1971, it has developed several plans related to human development. In 2020, the country’s population reached 9,282,041, which is expected to exceed 10.5 million by 2030 (Ellil, 2021). The country’s development, progress, and prosperity, particularly through the use of global competitiveness indicators have been an emerging area of research for consumers and scholars in low-income societies. The unique theme of this research is an advance to the literature as it integrates several key concepts like innovation capability in education sector that has never been addressed in previous literature. The research gap being filled through this research is another major advance in this study. Current study is novel in terms of focus on HDI and innovativeness in education sector. Specially, such studies in middle eastern context are very rare that makes its unique contextual contribution.

The current research is innovative in terms of minutely focusing on education sector of one country to explain that how it is linked with HDI and competitiveness and how ultimately it helps in achieving national goals. However, the current research was conducted on the role of innovation capabilities in human development competitiveness in the Education Sector in UAE. Finally, the current study has been based on the Human Capital Theory. It is defined as “know-how, information, relationships, and general relationships, and general capabilities that individuals bring to bear on behalf of the form through the employment relation” (Fényes and Mohácsi, 2020). It is determined by economic studies and has been a primary driver for education policies and development. Based on this theory, current study advanced the literature by taking a stance to explore the linkages between innovation capabilities, human development, and education sector adoptability of innovation and technology. Furthermore, the current study aims to achieve the following objectives:

• To clarify the role of innovation capabilities in achieving and improving the UAE’s human development indicators.
• To identify the interrelationship between the UAE’s innovation capabilities and human development indicators in the education sector.
• To understand the weaknesses and strengths of UAE’s human development indicators in education.

## Literature review

The current research focuses on the role of innovation capabilities in human development competitiveness in the education sector in the UAE. The education system is frequently regarded as a necessary base for the development of human capital since it is viewed as an expectation of future production rather than a means of consuming resources. Studies linking innovation capabilities to human development in the education sector are scarce in the literature. Especially current research grounded in Human Capital Theory contributes to the case of the UAE education sector for developing and low-income countries.

### Human Capital Theory

The current study has been based on the Human Capital Theory. It is defined as “know-how, information, relationships, and general relationships, and general capabilities that individuals bring to bear on behalf of the form through the employment relation” (Fényes and Mohácsi, 2020). Institutions and states accept the human capital idea as a tool for educational change (Holborow, 2018). It comes with a specific aggregate at production levels and is included in human capital. Human capital is held by the person rather than the company, and people contract their assets.

Human capital is the most critical asset in education and skill development (Galiakberova, 2019), therefore mobilizing and translating resources into human capital is a good idea. To engage its employees, the human resource strategy for human capital development encompasses professional development, career opportunities, and organization development (Sanni et al., 2013; Fényes and Mohácsi, 2020). According to White (2021), the primary problem of human resource development is to achieve a balance between educational content and changing constantly market demands.

**FIGURE 1:**
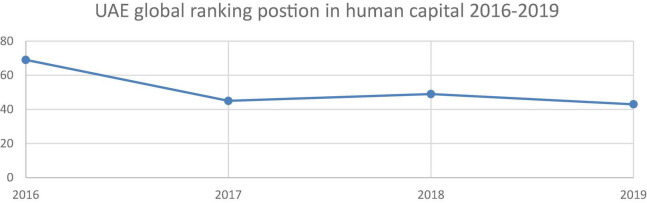
Human capital in UAE (2016–2019) (Index, 2020).

The global competitive positions that the UAE achieved in the human capital index from 2016 to 2019, data as displayed in Figure 1, show the UAE ranked 69th in 2016. This could be considered as a lagging position when compared to those of developed countries that are concerned with human capital. For instance, Singapore ranked 1st in the human capital development index for the year 2018, with a score of 0.88 points, while the UAE scored 0.66 points, a difference of 0.22. However, there was a gradual improvement in the UAE’s global position from 2017 to 2019, where it achieved 43rd after launching several initiatives to develop human capital, including the provision of integrated healthcare, attention to the development of education, the empowerment of job opportunities, and the increase of the rate of per capita income of GDP.

Human capital is an important element that serves to highlight the need for achieving a balance between human capital and sustainable development by enhancing innovation and developing research, thereby achieving sustainable human development (Foss and Laursen, 2012). Human capital positively impacts innovation and entrepreneurship, and enhances competitiveness due to its ability to innovate and improve competitive productivity. Moreover, it accentuates the need for continuous concern for human development and is essential for developing entrepreneurship and development projects (Vixathep, 2017). Governments often aspire to increase human capital investment and develop their intellectual side, as well as promote sustainable human development by focusing on building dynamic capabilities (Vixathep and Phonvisay, 2019; Vargas-Hernandez and Gonzalez, 2020). Additionally, there is a positive relationship between the number of patents and technological development, while a negative relationship exists between creators, employment growth creativity, and knowledge development (Fényes and Mohácsi, 2020).

### Innovation capabilities in education sector overview

To characterize innovation capabilities, Aljawarneh (2020) employs the terms innovation, adaptable, and absorption capacities. Elrehail et al. (2018) claim that product selection and corporate simulation are some of the main strategic stages in the process of innovation. From the perspective of corporate strategy, entrepreneurial and innovation competencies can be considered as a component of innovation capability. Oke and Fernandes (2020) define innovation capability as the ability to shape and manage a variety of capabilities. Other researchers have defined innovation capabilities as “innovativeness, new product development, and processes intellectual capital” (Elrehail et al., 2018; Aljawarneh, 2020).

Innovation capabilities in education can be described as the growth of a person’s cognitive, affective, and psychomotor domains and abilities in order for them to operate and perform optimally in society. Individuals must be assisted in maximizing their intellectual, social, and behavioral talents in order to benefit themselves and the entire society. As a result, Zhang and Hartley (2018) propose that the principle of higher education is the unified innovation of an individual’s overall, intellectual, devotional, and cultural abilities so that he or she is prepared to provide valuable service to humanity; rest assured that “education is the acquisition of knowledge, the aggregate of all processes through which a person develops the ability, skills, attitudes, and other forms of positive behavior in the society in which he lives (Vixathep, 2017)”.

**FIGURE 2:**
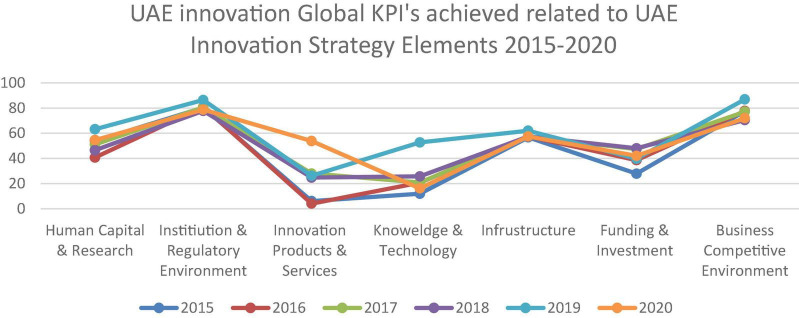
Global innovation in UAE. *Source*: (Index, 2020).

Figure 2 explains the comparative analysis of the innovation capability of the UAE as compared to global innovation competitiveness. Education improves a person’s character and allows him to grow his nature in such a way that he may contribute effectively to the growth of the nation to which he contributes (Foss and Laursen, 2012; Oke and Fernandes, 2020). Prior literature shows the theoretical research on innovation capabilities and performance in Vietnam, but they cannot fully understand the relationship between innovation capabilities and strategic performance (Vu, 2020). Some researchers examine the opportunity for human development in Qatar. And compares their challenges and innovation strategies with a sustainable environment (Waheed et al., 2015, 2017; Mohamed et al., 2022). However, current research is incremental to focus solely on the education sector and its linkages with innovation capabilities.

### Innovations capabilities in education sector UAE

The scope, variety, and development of educational programs and institutions in the UAE have grown significantly (Abdallah et al., 2012). According to the CIA World Fact Book, the United Arab Emirates has a demographic of around 5.6 million people and is ranked 112th in the world and the largest (AlNuaimi et al., 2021). In the United Arab Emirates, over 14% of the people are college-aged. Because of the political unrest and safety issues in the remainder of the Middle East area, UAE has become a popular education destination for MENA students who attend higher education (Jose and Chacko, 2017).

The UAE’s financial dynamism, along with its welcoming attitude toward expatriates and service for diversity, has fostered an influx of foreign educational providers from both the western and eastern. The bulk of universities in the Western world are from the United Kingdom, America, France, and Australia (Ahmed and Alfaki, 2013; AlNuaimi et al., 2021). In addition, 50% of the population is between the ages of 20 and 59. Because of the lengthy relationship between India and the UAE, as well as the closeness of the two nations, the UAE has a great chance to attract students from Asia (Moè, 2016). In addition, the UAE achieved a global innovation ranking of 34th and 1st within the Arab world in 2020 (Dutta et al., 2020). However, the UAE must still overcome certain obstacles and challenges it wish to be a top-10 tier HDI country, such as Norway, Ireland, Switzerland, and Hong Kong. Indeed, the UAE must use both tangible and intangible innovation capabilities to improve and promote its HDI requirements (Alfaki, 2018).

In terms of the factors mentioned above, the UAE’s aging population, along with cuts in government support for higher education in Western countries, has prompted several European and North American institutions to establish operations there (Jose and Chacko, 2017). While colleges from the West and East alike are motivated by the prospect of increased money, universities constructing campuses in the UAE do not experience the same population decline at homes as their regional peers. Indian colleges have been able to avoid the Indian government’s rigorous enrolment quotas based on facilities and job shadowing at home by building campuses in the UAE (Ellil, 2021). Furthermore, the authorities have no control over the tuition rates paid by these universities for their activities from outside the country. Thus, environmental and legislative, as well as competitive suitability, is attracting more educational brands as well as consumers from lower income societies to gain a quality education in UAE education market, which provide equally competitive education opportunity but on cheaper prices as compared to Australia, United Kingdom, Europe, and North American education markets. This study is incremental to highlight the innovation capabilities potential in the education sector which may reflect in a better HDI score for UAE to improve its ranking in the global competitive index.

### Role of innovation capabilities in human development competitiveness in the education sector

**FIGURE 3:**
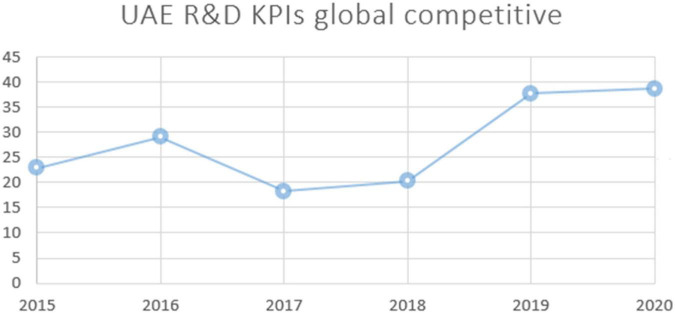
Innovation technology in UAE (2015–2020) (Index, 2020).

Scholars appeared to reach a common definition of the concept of human development in the 1990s. It has been defined as a multi-faceted process that aims to provide for the basic needs of humans over periods of time. These needs include a healthy and sustainable life, continuing education, and ensuring material stability (Al-Odeh, 2020). However, the United Nations Development Program (UNDP) defines human development as the improvement of life conditions by providing people with basic needs, allowing them to choose their potential capabilities, and practicing a happy life through receiving healthcare, quality education, and equal economic participation. Thus, the concept of human development refers to the basic motives for achieving individual and societal well-being, forming human capital and caring for and developing all aspects of life (Wang et al., 2019). The 12 components of competitiveness are “(1) Institutions, (2) Infrastructure, (3) Macroeconomic environment, (4) Health and primary education, (5) Higher education and training, (6) Goods market efficiency, (7) Labor market efficiency, (8) Financial market development, (9) Technological readiness, (10) Market size (11) Business sophistication and (12) Innovation” (Gallagher, 2019; Hamid, 2019). As explained in Figure 3, innovation technology adoption and comparative analysis of UAE with global market standards provide a good picture of the contextual importance of this research for low income and developing world trying to improve innovation human development through education.

UAE’s educational standards and higher education skills’ employment market competitiveness are still lagging below, both nationally and internationally. Education-related topics such as the vital role, school administration, educational attainments, the higher education system, instructional achievements evaluation, and others are frequently addressed (Gallagher, 2019). This is due to the fact that education is the primary system capable of causing societal change. A young generation will emerge as a result of education, with new and better ideals (Ziauddin et al., 2010; Cepeda and Arias-Pérez, 2018). The younger generation is a feature of user capabilities for a range of cultural and historic outputs. Most states’ attempts to devote their funding resources to establishing a high-quality education system will decide the nation’s future success. It will also influence how a country’s standing in the global competition will be determined. Some improvements for innovation capabilities in the education sectors of UAE are below. Firstly, it necessitated ongoing development or enhancement of present workflow to generate good initiatives that could remain competitive in the market for at least the next 2–3 years. Secondly, an institution could use internal learning or external knowledge to research for the next generation, based on the present items or technology. Finally, an organization should exhibit innovative process systems that include a continuous cycle of creativity, digestion, and re-innovation rather than relying on the operation of basic knowledge, which causes the new information to quickly become obsolete and lose its value as a source of innovation (Foss and Laursen, 2012; Hamid, 2019; Vixathep and Phonvisay, 2019). The main element in building collective innovative capital is government supportive and competitive pressure which helps to foster an innovation climate in the newly emerging education market. Such resources are supported by authorities in UAE by establishing education cities in almost every state of UAE. Thus, the current study is an advance to the body of literature to shed light on the emerging potential of the UAE’s education market for lower income societies in South Asia, Africa, and middle-eastern countries.

### A case of UAE education sector comparative analysis with top performers in human development index

The HDI is one of the most prominent annual reports launched by the UNDP and includes different standards for measuring human well-being and the extent of countries’ capabilities based on statistical data (El-Sholkamy and Fischbach, 2019). The HDI focuses on health, knowledge, and living standards in order to measure and enhance positive competitiveness (Bucher, 2018). The program places economic growth among its priorities and has a primary pillar of human development despite economic hardships, healthcare issues, and social factors causing high unemployment (Arshed et al., 2022). HDI includes a measure of the extent to which countries achieve levels of living development compared to their populations, as well as the rate of the continued sustainability of healthcare, education, economic growth, the environment, and societal well-being (Kozyr et al., 2018). Whereas there is nothing much published about HDI in the research in education sectors with HDI as a title search and the little that has been written has focused on the refining of HDI (Ilechukwu et al., 2014; Qureshi et al., 2020). This research is incremental to fill this gap in the literature and bring key insights into scholars and policy institutions.

The HDI also focuses on measuring the rate of knowledge and education in terms of countries’ populations and the per capita share of their GDP. This seeks to motivate countries to foreground education and its quality outcomes and provide the appropriate infrastructure that contributes to education’s ease of access, thereby furthering human development (Hamid, 2019). In fact, the HDI report reflects countries’ developmental progress and aims to continuously motivate them to improve this progress in accordance with the report’s results. This leads countries to reconsider their plans and policies, and how to face current challenges and development in line with the development plans of the United Nations (Bucher, 2018).

**FIGURE 4:**
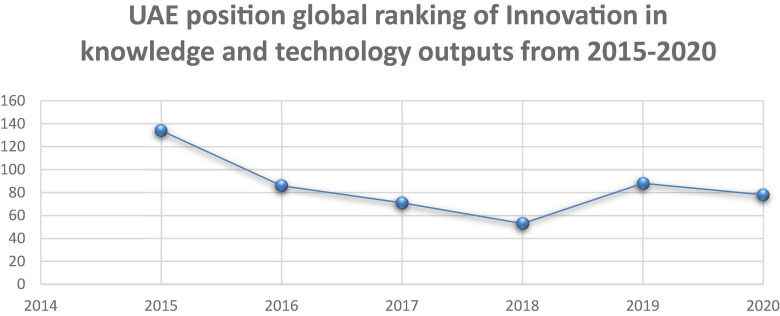
Global competitiveness index (2015–2020) (Index, 2020).

Figure 4 shows the competitiveness index on R&D achieved by the UAE between 2015 and 2020, where the results were measured in terms of consensual improvement. The UAE achieved 22.9 points in 2015, which gradually increased to 29.1 in 2016, but decreased between 2017 and 2018 by 18.1, resulting in 20.3. However, it increased significantly to 38.7 in 2020—a difference of 15.8 points compared to 2015. Based on data from the UAE’s Federal Competitiveness and Statistics Center (FCSC), 33.3% of total R&D workers were female in 2018, which increased slightly to 33.35% in 2021. The 55% of specialists interviewed were highly confident in the importance of R&D as one of the innovative possibilities that can improve the value of the HDI scores. Moreover, they confirmed that R&D is the essential tool for producing human development-enhancing knowledge and helps in employing its applied outputs for improving society’s quality of life. Accordingly, these findings are compatible with Arshed et al. (2022), and confirmed governmental reliance on R&D as an innovative ability for developing the health, educational, and economic sectors, thereby improving HDI scores. Further, 15% of them argued for its contribution to developing healthcare, education, and economics whose effects may be reflected in the country’s global leadership in terms of the HDI.

### UAE demographic trends

Firstly, the UAE’s demographics are special in terms of citizenship ratios, which have an impact on education and other aspects of living. In several aspects, such demographics are essential to any debate about education. Emirati individuals are often the only ones who have access to free, government-sponsored education. But those who are not citizens of the Emirates must pay their education fees (Ellil, 2021). As a consequence, Emirati citizens are nearly entirely represented in public education, which follows the UAE Ministry of Education’s curricula. Considering the country’s extremely unbalanced demographics, educational institutions sprouted up to meet the needs of the UAE’s more than 200 nationalities, offering a variety of curriculum designs, including American, Australian, British, Canadian, French, German, Indian, Pakistani, and international hybrids of these. Another feature of the country’s atypical demography is the substantial use of outside knowledge in the school system’s development (Jose and Chacko, 2017). In today’s industrialized countries, education is a juxtaposition of local and international, traditional and technological, indigenous and acquired. Education in the UAE, possibly more than in other countries, reflects modern industrialization and criterion, as evidenced by the expansion of foreign private schools and private higher education. Furthermore, public education in the UAE takes place in a fast-paced, ever-changing environment. Despite having one of the highest-performing educational systems in the Arab world and despite government spending on education (Gallagher, 2019).

The fact that men are often employed in hard-labor employment in UAE’s huge infrastructure renovation is the key reason for this less-educated, male-dominated non-UAE community. Over the last century, the UAE’s higher education has seen considerable changes and advancements (Galiakberova, 2019). Unfortunately, baseline measures reveal that UAE youngsters’ academic accomplishments lag behind those of their modern counterparts. Pupils in the UAE looked to have just rudimentary reading skills, similar to those in Qatar, and the number of students with only rudimentary reading skills was approximately five times greater than in experience and understanding nations (Jose and Chacko, 2017). The UAE community is relatively well-educated, with the majority of males having completed secondary school; however, UAE females are more likely to seek higher education. UAE expats, on the other hand, have a more limited educational background, with the majority of men only receiving an elementary education (Deffinika et al., 2021).

### Trends in the UAE education system

The great leader of the UAE, Sheikh Zayed bin Sultan Al Nahyan, emphasized that “the real asset of any advanced nation is its people, especially the educated ones, and the prosperity and success of the people are measured by the standard of their education”, which residents take great delight. While the UAE’s higher education is still new, the government has taken steps relentlessly in just a few brief years to make a system that meets the demands of the native and overseas populations (Ellil, 2021). The deployment of substantial cash for learning and public growth has backed up the stated intention. Furthermore, with increased attempts to determine consistency, there has been a significant pushing force to move the system of education to a strong position in the worldwide domain. It is necessary to offer context for the UAE’s educational ambitions and accomplishments in the quality assurance arena by first reviewing the country’s past and the development of its education sector (Hamidi and Zandiatashbar, 2019).

Educational systems for innovation have developed in many sectors, especially in the concept of the institutional theory of innovation, which has become more participatory by society. Moreover, it has contributed to the emergence of more effective innovative technologies to instigate a change in society’s quality of life and ability to face such developmental challenges as transportation, pollution, food security, public health, and education (Deffinika et al., 2021). The dynamism of technological innovation systems has demonstrated the ability of mutual interaction between all sectors and society, which impacts global competitiveness indicators, and contributes to the provision of modern technology for integrating services and infrastructure with innovative socio-technical models (Gallagher, 2019).

The problem statement of this research sheds light on the gray area where limited research has been conducted to explain the linkages between innovation capabilities and improvement in human capital indicators. Especially the role of human development indicators in education sector is very scarcely investigated in literature. In the context of UAE, it becomes more vital to test these theoretical notions on evidence from UAE. To date, none of such studies are found in literature considering these constructs in UAE context.

Based on all the above sections and academic debate, current research attempts to propose the following research questions to be explored and tested:

**Research Question 1**: *Does Innovation capabilities have a positive relationship with achieving and improving the UAE’s human development indicators?*

**Research Question 2**: *How UAE’s innovation capabilities and human development indicators are reflected in the UAE education sector?*

**Research Question 3**: *UAE education sector requires a strategic uplift using its weaknesses and strengths to achieve the desired position in HDI?*

## Research methodology

This study uses primary data gathered from reviewing the concepts and perspectives found in the existing literature. Following the qualitative research design and data were collected through interviews with 70 experts and leaders working in this field of HDI and innovation capability development in the UAE to more comprehensively recognize the main dilemmas involved and to shed light on gray areas. Overall, the data were collected for a bigger research project; however, this study only focused on the interview questions and answers related to innovation capabilities, HDI in the education sector to remain focused on study objectives and to answer the research questions that emerged in the literature review section. The main purpose of conducting interviews is first to understand the weaknesses and strengths of UAE’s human development indicators in education and second to clarify the role of innovation capabilities to achieve and improve the UAE’s human development indicators. This allowed us to verify or reject the research questions and identify the relationship between innovation capabilities and HDI. Moreover, it used a case study focusing on UAE human development key performance indicators (KPIs) based on previous reports from 2014 to 2020 to thoroughly analyze the strengths and weaknesses of UAE’s HDI related to education, and understand the gap between the UAE and more advanced countries. Additionally, the research depends on the secondary data collected from articles, journals, books, and websites and the data search ranged from 2014 to 2020.

### Interviews

The current study used semi-structured interviews with the UAE’s HDI experts related to education sectors, and data were collected through interviews with 70 experts and leaders working in this field in the UAE. Firstly, validate the presence of the stated human development economic, demographic, and educational imbalances. Secondly, based on the suggestions of interviewees, divide these gaps into broad themes. Thirdly, identify the future gaps and challenges and lastly give recommendations. This study used semi-structured interviews through a technique in which open-ended questions provide some structure while allowing interviewers enough latitude to provide fresh insights as presented in Appendix 1. Moreover, semi-structured interviews provide a depth investigation of the human development of the education sector in UAE and highlight the issues and challenges which occur in the education sector. Examples of the questions which are involved in the interview are state the Human Capital Theory, UAE innovation capabilities, prior literature about human development in the education sector, and the main purpose of the study. Each interview took at least 30–45 mins and 4–6 experts were included in a panel and answers were recorded in an audio file with the permission of experts; later transcripts were obtained from professional experts and used for content analysis.

### Participants

Using purposive sampling, we collected 70 participants’ interviews with a balance gender distribution and a wide range of background qualifications. All participants were contacted through personal references of one of the study authors to participate in this study. In this interview, we capture multiple perspectives about human development in the education sector. Interviewees were chosen based on their professional and relevant expertise, as well as their willingness to share their thoughts on the UAE’s efforts to transform its economy into a knowledge economy and the country’s human development requirements and concerns. More precisely, we chose policymakers and practitioners from the different education sectors’ teachers with relevant leads to better as those with appropriate information and expertise in the human development index. We were able to get an in-depth insight of the interviewees by using a purposive sampling approach. Some of the participants had a lot of expertise and were decision-makers in their areas; over half had experience in two or more sectors, which gave the study a lot of depth. All study participants gave their informed permission for voluntary participation and researchers assured them of strict anonymity of personal information and demographics that none of their departmental or personal identification with responses will ever be shared with any third party. All participants were in higher positions and experts in the English language; so interviews were conducted in English. The study data were analyzed to check demographics, content analysis, excel graphs, and chart, and bar presentations were conducted to produce study results.

## Results

### Demography

The results were evaluated using SPSS, according to the current study conducting interviews of experts in education sectors in UAE; thus, all demographic variables are presented below in Table 1.

**TABLE 1:** Demographic details.

Demography	No. of responses	%
Male	45	64
Female	25	36
25–35	50	71
Above 35	20	29
Bachelors	47	67
Master	23	33
Resident of UAE	50	71
Non-resident of UAE	20	29

Table 2 shows the results of the technological infrastructure readiness of KPIs for the percentage of the availability of information on government agencies between 2012 and 2020 that affect human development and societal well-being. The table shows that the percentage of society’s access to information related to government agencies was 15.66% in 2020. Depending on the perspectives of the specialists in the field of human development and competitiveness, 60% approved the positive relationship between innovative technology and improving HDI, while 10% argued for several reasons, such as the rapid technological developments which can vastly contribute to improving areas of human development. Moreover, this finding accords with Deffinika et al. (2021), who found that the development of innovative technological systems according to the concept of institutional theory has become more participatory and integrative, which has served to significantly improve society’s quality of life and the enhancement of human development.

**TABLE 2:** Readiness of technological infrastructure in government sectors (during 2012–2020).

Readiness of technological infrastructure in government sectors (during 2012–2020)					
Components	2012	2014	2016	2018	2020
Governments sectors	30%	19%	14%	13.80%	15.66%

Table 3 shows the results of the Federal Competitiveness and Statistics Center, UAE Government Portal, Digital, UAE Government budget and expenditure UAE (FCSC) and Ministry of education (MOE) open data, 2020 World Bank, IBRD, World competitiveness ranking 2020–2021, UNDP (HDI) reports 2014–2020, and Global Innovation Index (GII) Reports 2014–2020. Based on the data issued by the Federal Competitiveness Authority 2019–2021 and United Nations annual reports of HDI refers that the percentage of government expenditure on education, and total % GDP was stable at 3.1% in 2021. The total public expenditure on education per capita was 1375$ in 2021, while the total expenditures on education (AED Million) was 23,190.2 in 2016; however, the number was slightly decreased to 23,143.04 in 2019. In leadership KPIs for education, QS–University ranking average (global score 0–100) was 35.8 in 2021. The net influx of International students has been 7.1 in 2014; however, it increased to 7.2 in 2020. Also, the percentage of tertiary inbound mobility was maintained by 48.6% in 2021. The student’s performance in reading, science, and math (PISA) was stable at 433.5 in 2021; however, the percentage of students who are not low achievers – PISA was 42.5% in 2021. The percentage of the population having secondary education was 72.2% from 2010 to 2017, while it increased in 2020 to 99.7%. The percentage of female enrollment in tertiary education was 69% in 2020. Critical thinking in education achieved 5.09 in 2019. However, the rate of graduate skills reached 5.08 from 2019 to 2020. The educational level of the adult population was 54% in 2020. The percentage of uneducated (Female) was 13% in 2020 while the UAE did not achieve the Nobel Prizes, whether at the general or individual level. The gender parity in secondary attainment (distance from parity) was 0.20 in 2020. The education inequality, Gini co-efficient of education distribution among 15+ population, accounting for average years of schooling among the population was 0.22 in 2020. MOE providing technological services in the educational environment, the percentage of primary and secondary schools equipped with the Internet, has reached 100% from 2014 to 2019. The individual digital and technologies skill in UAE was achieved to 7.7 in 2020, while it was increased to 8.1 in 2021. MOE developed the smart educational environment, which benefited 25,000 teachers and 300,000 students. The percentage of schools equipped with computers is 100%. MOE launched Mohamed Bin Rashid Smart Education Project to include 150 schools. MOE established virtual education initiative in 2019 to include 25% for normal study cases, 25% for exceptional study cases, and 100% for comprehensive education, also it included 335 pre-primaries and the percentage of teachers who attended 98%. The percentage of schools that applied virtual education was 99%. MOE launched 1,320 virtual learning communities with allocated 23,300 teachers. The number of virtual education workshops has reached 276. The number of virtual educational platforms provided for applied technology, innovative engineering, computing, and artificial intelligence by reaching 20.

**TABLE 3:** Innovation capabilities inter relation with human development index (HDI) key performance indicators (KPIs) of education (2014–2020).

Innovation capabilities	Innovation capabilities inter relation with HDI KPIs of education (2014–2020)					

UAE National Innovation Strategy 2015 (Education sector)	Funding KPIs for education					
		
	Education components	2016	2017	2018	2019	2020
	Government budget allocated for education (AED Billion)	6.526	10.2	10.4	10.14	10.41
	% of federal budget allocated for education	13.40%	20.50%	20.20%	16.80%	14.80%
	Government expenditure on education, total % GDP	3.10%				
	Based on data issued by the Federal Competitiveness Authority 2019–2021 and United Nation annual reports for HDI refers that the percentage of government expenditure on education, total % GDP was stable by 3.1% in 2021. The total public expenditure on education per capita was 1375$ in 2021, while the total expenditures on education (AED Million) was 23,190.2 in 2016, however, the number was slightly decreased to 23,143.04 in 2019					

	**Innovation capabilities inter relation with HDI KPIs of education (2014–2020)**							

	**Leadership KPIs for education**							

	**Education components**	**2014**	**2015**	**2016**	**2017**	**2018**	**2019**	**2020**

	QS – University ranking average (score 0–100)	28.8	34.5	32.5	28.9	29.3	31.2	32.8
	% Tertiary inbound mobility	39.80%	44.60%	44.80%	46.90%	48.60%	48.60%	48.60%
	Students performance in reading, science and math (PISA)	435.3	432.6	432.6	433.6	433.6	433.9	433.5
	QS – University ranking average (global score 0–100) was 35.8 in 2021. The net influx of International students has been 7.1 in 2014; however, it was increased to 7.2 in 2020. Also, the percentage of tertiary inbound mobility was maintained by 48.6% in 2021. Regarding the students’ performance in reading, science and math (PISA) was stable at 433.5 in 2021; however, the percentage of students who are not low achievers – PISA was 42.5% in 2021							

	**Leading operations and services KPIs for education**							

	**Education components**	**2014**	**2015**	**2016**	**2017**	**2018**	**2019**	**2020**

	No. Schools (Government and Private)	1215	1215	1230	1226	1219	1262	1257
	No. Government schools	673	673	667	659	639	619	600
	No. Private schools	542	542	563	567	580	643	657
	No. Higher Education Institutions	74	74	74	76	76	159	159
	Expected years of study	13.3	13.3	13.3	13.6	13.3	14.3	13.6
	Average years of schooling	9.5	9.5	9.5	10.8	11	12.1	15.7
	Literacy rate	93.50%	97.50%	97.50%	97.50%	97.50%	97.50%	93.80%
	% Teachers qualified and trained in primary education	100%	100%	100%	100%	100%	100%	100%
	% of students from teachers at the primary education	25%	25%	25%	25%	24.50%	24.50%	24.50%
	% of students from teachers at the secondary education	14.3	11.5	13.3	11.2	9.5	9.5	9.5
	No. students for per teacher in primary education	16	19	25	25	25	25	23.2
	% of enrollment in pre- education	79%	92%	82%	82%	98.00%	98%	98.00%
	% of enrollment in primary education	108%	107%	101%	101%	90%	90%	108%
	Adjusted net enrollment rate, in lower secondary education	94%	94%	94%	98%	98%	95.80%	92%
	% of enrollment in secondary education	96%	96%	96%	96%	98%	98%	94%
	% graduates (science, math, engineering, manufacturing, construction industries) at the higher education level	14.20%	13.90%	14.40%	19.70%	22.00%	22.00%	27.70%
	% Graduates in science and engineering	26.00%	26.00%	20.40%	20.40%	22.00%	22.00%	27.70%
	%Enrollment in tertiary	22%	22%	37%	37%	33%	33%	60.80%
	Educational attainment, at least completed lower secondary, population 25+ years	63%	63%	71%	78%	81%	84.10%	87.40%
	Educational attainment, at least completed primary education, population 25+ years	74.80%	74.80%	83%	87%	91%	93%	93%
	Educational attainment, at least completed secondary, population 25+ years	64.30%	67.70%	69%	69%	69%	82%	82%
	% of people have tertiary education	22%	22%	22%	37%	37%	34%	34.80%
	Tertiary Education (score 0–100)	N100	68.3	49.2	59.1	56.6	57.5	66.4
	Percentage of students from teachers at the primary was 25% from 2014 to 2017, while it decreased to 18.8% in 2021, however the proportion of students to teachers in secondary was 9.4% in 2020 and it increased to 10.6% in 2021, and in the tertiary education was 18.4% in 2020 and it increased to 18.6% in 2021. The number of educational and vocational training institutions 23 in 2021. The rate of literacy achieved 100% in 2021, while the rate of illiteracy achieved 6.8%. The percentage of graduates in science and engineering was increased to 31% in 2021							

	**R&D KPIs for education**							

	**Education components**	**2014**	**2015**	**2016**	**2017**	**2018**	**2019**	**2020**

	Government spending on R&D	6%	9%	9%	9%	10.30%	10.30%	10.30%
	Gross expenditure on R&D % GDP	0.50%	0.50%	0.70%	0.90%	1.00%	1.00%	1.30%
	Research and Development (R&D) score 0–100)	19.9	22.9	29.1	18.2	20.3	37.7	38.7
	Research collaboration between University and Industry (score 0–100)	63.2	62.1	62.1	58.5	57.9	55.7	59.9
	Scientific and technical articles/bn-PPP$ GDP (score 0–100)	5.1	2.7	3.2	3.5	2.9	3.1	3.9
	Human capital and Research (score 0–100)	62.1	53.9	40.7	51	46.5	52.4	54.6
	Human capital and Research in the education (score 0–100)	66.3	70.6	43.8	75.6	62.5	61.9	58.6
	Percentage of female researchers was 33.3% in 2020, while it increased to 39% in 2021; however, 92 students from UAE University obtained researcher license in 2019. No. of researchers, FTE/MN, POP was 2,406.6 in 2018–2019, while it increased to 2,378.9 in 2020. The No increased to 3,145 in 2021. The scientific research legislation was 7.2 in 2020, while it increased to 7.7 in 2021. The UAE government is interested in supporting researchers, as it launched (Nation Fund) it’s a national platform for funding scientific research through three main programs (The Emirati Research – Applied and Development Research – Research Grants). The indicator of the scientific research institutions emergence achieved 0.015 in 2019, and the No of scientific research publications was 170.6 in 2019. The student mobility outbound achieved 1.31% in 2021. However, the international mobile students (men and women) from abroad studying in a given country (in tertiary education). Data can refer to the school or financial year prior or after the reference year achieved 8.4% in 2020							

**Human capital KPIs for education**							

**Human capital**	**Education components**	**2014**	**2015**	**2016**	**2017**	**2018**	**2019**	**2020**

	Expected school years (Male)	12.9	12.9	12.9	13.4	13.4	14.1	14.1
	Average years of schooling (Male)	8.5	8.7	8.7	9.7	9.8	12.4	12.4
	Expected school years (Female)	13.9	13.9	13.9	14.3	14.3	14.8	14.8
	Average years of schooling (Female)	9.9	10.6	10.6	11.9	12	11.7	11.7
	% of enrollment in pre- education (Female)	108%	90%	108%	108%	108%	95%	95%
	% of enrollment in primary education (Female)	98%	107%	97%	97%	97%	98%	98%
	% of enrollment in secondary education (Female)	94%	94%	94%	94%	94%	92%	92%
	Educational attainment, at least completed primary education, population 25+ (Female)	85%	85%	85%	85.70%	87.60%	90.70%	90.70%
	Educational attainment, at least completed primary education, population 25+ (Male)	74%	74%	74%	91%	93%	94.40%	94.40%
	% Population at least have secondary education, aged 25 and over (Male)	61.20%	65.70%	65.70%	65.70%	65.70%	81%	81%
	% Population at least have secondary education, aged 25 and over (Female)	73.10%	77.40%	77.40%	78.80%	78.80%	76%	76%
	Educational attainment, at least completed upper secondary education, population 25+ (Male)	44.70%	44.70%	44.70%	68.60%	70%	71.60%	71.60%
	% of graduates from tertiary education (Female)	51%	54%	55%	51.80%	51%	55%	55%
	% graduates (science, math, engineering, manufacturing, construction industries) at the higher education level (Female)	17.30%	17.30%	17.30%	17.30%	43.50%	41.50%	41.50%

	**Education components**	**2014–2015**		**2015–2016**	**2016–2017**	**2017–2018**	**2018–2019**	**2019–2020**

	No. Teachers in public schools	25923		26208	27210	30667	32498	32498
	No. Teachers in higher education Institutions	7723		8249	6981	7297	8207	7916
	No. students in technical and government general education	984,942	1,063,177	1,076,464	1,107,905	1,126,701	1,140,532
	No. Students in technical education	9,734	8,315	9,061	10,417	9,230	8,505
	No. Students in government education	274,393	277,733	280,841	287,725	288,794	286,550
	No. Students in private education	687,214	755,882	777,459	793,295	810,537	826,993
	No. Students at home and elderly education	13,601	21,247	9,103	16,468	18,140	18,484
	No. completion of studies and graduation in higher education	24667	25285	22103	21644	22059	22059
	No. students dropped out in general education because marriage	19	12	30	32	13	13
	Percentage of population have secondary education was 72.2% from 2010 to 2017, while it increased in 2020 to 99.7%. The percentage of female enrollment in tertiary education was 69% in 2020. The critical thinking in education achieved 5.09 in 2019. However, the rate of graduate’s skills reached 5.08 from 2019 to 2020. The educational level of the adult population was 54% in 2020. The percentage of uneducated (Female) was 13% in 2020. While the UAE did not achieve the Nobel Prizes, whether at the general or individual level. The gender parity in secondary attainment (distance from parity) was 0.20 in 2020. The education inequality, Gini co-efficient of education distribution among 15+ population, accounting for average years of schooling among the population was 0.22 in 2020						

**Innovation knowledge KPIs for education**								

**Innovation knowledge**	**Education components**	**2014**	**2015**	**2016**	**2017**	**2018**	**2019**	**2020**

	Innovation knowledge and technology outputs value (global score 0–100)	14.3	12	20.8	20.9	25.7	22.2	16.2
	Citable documents H index	87	100	112	9.4	10.1	10.5	12.2
	Patent families (3), (2) + offices/bn- PPP$ GDP	0	0	0.1	0.1	0.1	0	0.1
	Percentage of knowledge transfer in the UAE between companies and universities reached 6.7 in 2020, while it increased to 7.08 in 2021. MOE launched an initiative with the aim of presenting the students innovative ideas. MOE sent 45 students to United States and Netherland with the aim of enabling them to learn about innovative tools in modern science. MOE launched innovation ambassadors with the participation of 200 teachers and 650 students from 2016 to 2018. MOE launched innovation competition for university students. Also, established the innovation pioneers award for students. Organized the National Science, Technology and Innovation festival with the participation of 498 students and 153 schools. The number of innovative student’s projects 400 from 2018 to 2020							

	**Education components**	**2015**	**2016**	**2017**	**2018**	**2019**	**2020**	

	No. Students participate an artificial intelligence and robotics competition	35	731	1437	2595	3020	7144	
	No. completion of studies and graduation in higher education	24667	25285	22103	21644	22059	22059	
	No. students dropped out in general education because marriage	19	12	30	32	13	13	
	MOE providing technological services in the educational environment, the percentage of primary and secondary schools equipped with the internet has reached 100% from 2014 to 2019. The individual digital and technologies skill in UAE was achieved 7.7 in 2020, while it was increased to 8.1 in 2021. MOE developed the smart educational environment, where it benefited 25,000 teachers and 300,000 students. The percentage of schools equipped with computers 100%. MOE launched Mohamed Bin Rashid Smart Education Project to include 150 schools. MOE established virtual education initiative in 2019 to include 25% for normal study cases, 25% for exceptional study cases and 100% for comprehensive education, also it included 335 pre-primaries and the percentage of teachers attended 98%. The percentage of schools applied virtual education was 99%. MOE launched 1,320 virtual learning communities with allocated 23,300 teachers. The number of virtual education workshop has reached 276. The number of virtual educational platforms provided for applied technology, innovative engineering, computing and artificial intelligence by reached 20							

Federal Competitiveness and Statistics Center, UAE Government Portal, Digital, UAE Government budget and expenditure UAE (FCSC) and Ministry of education (MOE) open data, 2020 World Bank, IBRD, World competitiveness ranking 2020–2021, UNDP (HDI) reports 2014–2020 and Global Innovation Index (GII) Reports 2014–2020.

Table 4 shows the results of the Federal Competitiveness and Statistics Center, UAE Government Portal, Digital, UAE and Global Innovation Index (GII) Reports 2014–2020. UAE’s global potions are ranked by their scours in educational sectors. It is clearly visible from various indicators that improvement in the education sector gradually has helped to improve the global rank, as well as the competitiveness of the education sector and its contribution to HDIs.

**TABLE 4:** Global ranking of education key performance indicator (KPI) Vs UAE.

NO.	Education KPIs	UAE globally position	Score	1st globally ranking	NO.	Education KPIs	UAE globally position	Score	1st globally ranking
1	Higher education achievement	47th	33.1	Kazakhstan	32	Enrolment In Secondary Education	131st	0.949	Multiple countries
2	Educational assessment PISA - Math	45th	434.9	China	33	Expenditure on education	94th	3.1	Namibia
3	Female researchers	39th	33.35	Venezuela	34	QS university ranking, average score top 3*	36th	32.8	United States
4	Pupil-teacher ratio (tertiary education)	42nd	18.4	Japan	35	Pupil-teacher ratio, secondary	33rd	10.5	Croatia
5	Scientific and technical employment	35th	3.8	United States	36	QS university ranking average score top 3 universities	33rd	35.8	United States
6	Total public expenditure on education	62nd	1.98	South Africa	37	Researchers	50th	3,145	South Korea
7	Citable documents H-index	61st	12.2	Multiple countries	38	Tertiary enrolment	60th	52.6	Greece
8	Adult literacy (%)	104th	93.8	Multiple countries	39	Female graduates	73rd	55.15	Tunisia
9	Expected year of schooling	86th	13.6	Australia	40	Population with secondary education	84th	18.2	Kyrgyzstan
10	Gross enrollment ratio (Education)	145th	67	Multiple countries	41	Quality of management schools	24th	5.21	Switzerland
11	Mean year of schooling	54th	11	Germany	42	Scientific journal articles	54th	2.33	Switzerland
12	Pupil-to-teacher ratio in primary education	92nd	24.52	Luxembourg	43	University ranking	36th	32.83	United States
13	Quality of education vocational training	25th	4.8	Switzerland	44	Education vocational enrolment	108th	1.89	Bolivia
14	Research institutions prominence index	59th	0.015	United States	45	Education facilities	52nd	55	Poland
15	School life expectancy	74th	13.6	Australia	46	Education outcomes	63rd	50	Multiple countries
16	Scientific publications	64th	170.6	United States	47	Scientific and technical articles/bn PPP$ GDP	97th	3.9	Denmark
17	Lower-secondary completion	76th	87.4	Multiple countries	48	Knowledge creation	104th	5.6	Switzerland
18	Pre-primary enrolment	69th	66.39	Peru	49	Access to quality education	28th	3.5	Japan
19	Primary education quality	64th	448	South Korea	50	Average quality of higher education institutions	28th	0.284	Multiple countries
20	Citable documents	48th	0.88	Monaco	51	Education inequality	83rd	0.22	Albania
21	Quality weighted universities	46th	28.8	United States	52	Education level of adult population	79th	0.54	Russia
22	Secondary school attainment (% of population)	75th	70.7	Multiple countries	53	Primary enrolment under 15 age	63rd	95	Norway
23	Illiteracy (%)	60th	6.8	Multiple countries	54	Secondary education quality	55th	460.69	Singapore
24	Student mobility outbound	36th	1.31	Cyprus	55	Secondary school enrolment	33rd	92.8	Kazakhstan
25	Research and Development (R&D)	28th	38.7	South Korea	56	Gender parity in secondary attainment (distance from parity)	116th	0.2	Multiple countries
26	Knowledge diffusion	71st	21.1	Ireland	57	Primary school enrollment (% of children)	58th	98.7	Ireland
27	Patent families 2+ offices/bn PPP$ GDP	62nd	0.1	Multiple countries	58	Women with advanced education (%)	73rd	0.69	Lithuania
28	Research and Development (R&D)	28th	38.7	South Korea	59	Women with no schooling	133rd	0.13	Multiple countries
29	Knowledge impact	72nd	21.9	Ireland	60	Nobel prizes	29th	0	United States

Federal Competitiveness and Statistics Center, UAE Government Portal, Digital, UAE and Global Innovation Index (GII) Reports 2014–2020.

Table 5 shows the results of Federal Competitiveness and Statistics Center, UAE Government Portal, Digital, and UAE and Global Innovation Index (GII) Reports 2014–2020. The human development and innovation indicators are the global strength of UAE achieved in 2020–2021 educational ranking. Enrolment in primary education, literacy rate, tertiary-level inbound mobility, international students, primary completion, and enrolment in primary education is ranked 1st position. Tertiary education ranked as 2nd position and research talent, % in business enterprise ranked as 3rd position and so on intellectual property receipts, % total trade ranked 19th and last position. These show improvement areas in UAEs education sector for better competitiveness in global HDIs ranking.

**TABLE 5:** Global innovation index Vs UAE.

NO.	Components	Education KPIs	UAE globally position	Score	NO.	Components	Education KPIs	UAE globally position	Score
1	Education	Enrolment In Primary Education	1st	1	12	Education	Knowledge absorption	16th	48.7
2		Literacy Rate	1st	1	13		Digital/Technological skills	17th	7.79
3		Tertiary level inbound mobility	1st	48.6	14		Graduates in Sciences	17th	27.73
4		International students	1st	48.5	15		Knowledge transfer	16th	6.76
5		Primary completion	1st	108.34	16		Scientific research legislation	14th	7.24
6		Enrolment In Primary Education	1st	1	17		Women with degrees	19th	47.4
7		Tertiary education	2nd	66.4	18		Skillset of graduates	14th	5.08
8		Education	17th	58.6	19		Pupil-teacher ratio (secondary education)	18th	10.6
9		Global R&D companies, avg. exp. top 3, mn $US	18th	67.6	20		Human Capital and Research	17st	54.6
10		Research talent, % in business enterprise	3rd	77.9	21		Graduates in science and engineering	15th	31
11		Intellectual property receipts, % total trade	19th	1	22		Population with tertiary education	17th	34.86

Federal Competitiveness and Statistics Center, UAE Government Portal, Digital, UAE and Global Innovation Index (GII) Reports 2014–2020.

## Discussion

The study findings revealed that innovative capabilities played an important role through their link to human development index (HDI). However, this link may be uneven based on the quality of the innovative ability and its connection and direct impact on improving competitive HDI. The importance of innovative capabilities, and the extent to which they are related to education, as well as their positive impact on improving HDI, has been subject to some debate. However, this identified the qualitative capacity of the innovation strategy in the educational sectors. This was evidenced by its major and comprehensively positive impact found on 11 axes of diversity between financial budgets and global leadership, society, services, and operations. In this regard, the study found that the innovation strategy contributes to supporting and improving 29 indicators for the educational sector. Thus, confirming research question 1 of this study. These findings also confirm the results of previous studies on the importance of applying the innovation strategy by focusing on human development, as well as the positive correlation between the innovation strategy and educational performance (Conceição, 2020). Further, the research also found the concern of prior research indicating that the adoption of the innovation strategy effectively contributes to enhancing indicators related to human development, especially by relying on the innovative technology used to provide educational services (Alfaki, 2018). The innovative capacity of human capital also has a positive impact on improving the HDIs in educational sectors, and through its association with 45 primary indicators as demonstrated. Also played an important role in terms of human development and engaging in innovation due to its use in helping achieve certain national objectives, such as the reduction of expenditure, procedural improvement, and the invention of new systems to promote human development. These findings also supported research question 2 of this study.

As per research question 3, the current study added to highlight that innovativeness in UAEs education sector is weaker as compared to other top performing countries on HDI. Another major weakness in education sector is the lack of capability development of academic staff in the UAE education sector. These weak areas need to be addressed by policymakers. One of the major strengths of the UAE education sector is diversity in population, as well as the regional central location for Asian and middle-eastern countries, which can be utilized for further policy developments. Based on interview results from the 45% of experts, human development agreed that human capital is the main part of innovation capabilities as it played an important major role to achieve HDI by focusing on the basic aspects of their life needs, such as educational levels, which is reflected on the HDI value ranking particularly in the quality of life; however, 25% of experts confirmed to realize that the human capital requirement is leading to increase the HDI and this must be done in a coordinated manner and cooperation with government agencies and institutions, also they assured that one of the most important pillars of attention to the human element is the process of participation and empowerment in society through increasing the quality of education outputs. So, these views were consistent with Holborow (2018) who demonstrated the importance of human capital investment and promoted the sustainable human development by focusing on building dynamic capabilities in providing an essence of quality of life factors infrastructure. The study has achieved the research questions/objectives aimed at the beginning to shed light on the linkages between innovation capabilities in education sector and HDI. The study also helped to highlight key weaknesses and strengths of UAE education sector for further integration and policy development to improve its rank in HDI and Global innovation index.

### Contribution to the field

Current research is significant due to its several important contributions to the field of innovation management, HDI, and global innovation index. Current research is also incremental for education sector by explaining how education sector goals can be aligned with the goals of national agenda for better ranks and ultimately what contribution the education sector may have to global sustainable development goals. Integration of unique innovation theories into a novel framework will also help the field in further growth. Scholars working in these constructs may get key leads for future studies to further extend the conceptualizations used in this study framework.

### Theoretical implications

Theoretically, this research contributes to the role of innovation capabilities in human development competitiveness in the education sector in the case of UAE’s emerging education case. The positive role of focusing on Human Capital Theory is an innovative ability that contributes to improve the results of competitive HDIs in the aforementioned fields, and that human capacity positively impacts educational sectors. This research theoretically integrated multiple conceptualizations into a scholarly framework. Human capital theories, innovation theories, and educational leadership and development theories may find further growth and integrative potential in future studies based on the theoretical integration of this research. Also, the study demonstrates other innovative capabilities, such as R&D, knowledge innovation, and technological innovation, that have somewhat equal impact on HDIs hence providing room for further theoretical exploration in all technology adoption and technology resistance, as well as innovation acceptance and innovation resistance domains of research. Moreover, these indicators may have an impact related to other axes concerning human development. As a result of this collaboration, new avenues for human development in education sectors research with theoretical assumptions have emerged for future studies. The major theoretical advance of this research is the integration of diverse ideas to bridge theory gaps among fields of innovation management, technology management, education leadership, and human development theories. The study is relevant to scientific community for bringing key insights about innovativeness of education sector and its contribution to overall economy and HDI of the country; thus pointing toward huge potential of education sector for achieving sustainable development goals nationally and internationally.

### Practical implications

Furthermore, this research provides policymakers, practitioners, and managers with relevant information in a variety of ways. To begin with, the current study shows that role of innovation capabilities in human development competitiveness in the education sector of UAE is vital and it has a reversible connection with innovative capabilities; the education sector becomes more adoptive to innovation, and resistance to innovation is overcoming. Contrarily, when the education sector has such capability, it produces a more vibrant human capital which in turn enhances the innovation capability of the national frame. Moreover, the indicators related to the educational sector show that the UAE has achieved positive results in both educational services and leadership. However, there are still some challenges to improve the total of these indicators, especially those related to research, development, scientific publishing, and knowledge management. In a conclusion, the researcher may assert that there is a link between educators’ innovation capabilities and human development toward educational technology. As a result, the more positive teachers’ innovation capabilities and strategies toward the educational sector are the more important factor. The developing and low-income societies may follow the improvement model of innovation capability development by the UAE education sector. Creating infrastructure support like education cities development is a major best practice that can be borrowed from the case of UAE. Another major policy insight for the developing world is the linkages between HDIs and education sector parameters that are evident from the findings of this research. So these economies that are striving to improve their HDI rankings may obtain key policy insights from the findings of this research. UAE policymakers may also get the benefit to improve education sector parameters where UAE is legging from top performers of HDIs. The practical managerial implications are mentioned above in detail. However, this research has social and informational aspects of contributions to highlight the importance and significance of education sector in any emerging economy. The study provided insights that for growth of any country on HDI, it must focus and develop their education sector.

### Limitations and future research directions

The present study, like all others, has significant flaws that need to be addressed in future research efforts. Firstly, this study contributes by developing recommendations that would likely help improve the UAE’s HDI results and advance its global position by focusing on innovative capabilities which can be linked to HDIs in educational sectors. Thus, generalizability of this research to other low-income and developing economies may be questioned. So, future research is recommended to adopt the comparative approach of two or more economies to bring more causal relationships. In future studies, societal awareness of the importance of indicators of competitive human development can be achieved through universities and educational institutions. Secondly, the data were collected through semi-structured interviews. In future studies, researchers can use a survey method or disturbed questionnaire and collect data. Thirdly, for more reliable results, researchers can use mixed-method search for more accurate results. Lastly, this study only uses the Human Capital Theory. In future studies, researchers can use multiple theories for more accuracy. This research was cross-sectional; so future longitudinal studies are recommended to find better causal relationships among investigated variables.

### Conclusion

Human capital is the one of the resources to build innovative capabilities that will facilitate the governmental development of the education sector. Moreover, education plays a prominent role in human development by achieving gains that impact the society’s development and that convince with the UAE’s Vision 2021 by focusing on several strategic priorities. UAE has developed a national agenda, including the addition of smart education devices to school curricula, and setting tests and assessing students’ skills and knowledge, such as the TIMSS and PISA exams in mathematics, sciences, and reading, the agenda also included a focus on increasing the pass rate in line with international rates. Therefore, it would be beneficial to focus on these indicators and reduce the gaps in the future by applying innovative practices and benefiting from innovative capabilities. Indeed, the study’s findings demonstrate that the government can prepare plans to reduce the HDI gap. The study also brings several key insights for theoretical and practical linkages between innovation capabilities, HDIs, and the education sector of any economy. Thus, highlighting the importance of educational leadership and technology adoption and innovative capabilities achieves sustainable development goals of the country.

## Data availability statement

The raw data supporting the conclusions of this article will be made available by the authors, without undue reservation.

## Ethics statement

Ethical review and approval was not required for the study on human participants in accordance with the local legislation and institutional requirements. Written informed consent from the patients/participants OR patients/participants legal guardian/next of kin was not required to participate in this study in accordance with the national legislation and the institutional requirements.

## Author contributions

SA worked on all major parts of this work. AA supervised and provided help in theory development and theoretical framework and worked through out the project in all sections. AL helped in data analysis. NS helped in improving the “Discussion” section and overall write up. SK helped in proof editing, argument building, and highlighting contribution. All authors contributed to the article and approved the submitted version.

## Conflict of interest

The authors declare that the research was conducted in the absence of any commercial or financial relationships that could be construed as a potential conflict of interest.

## Publisher’s note

All claims expressed in this article are solely those of the authors and do not necessarily represent those of their affiliated organizations, or those of the publisher, the editors and the reviewers. Any product that may be evaluated in this article, or claim that may be made by its manufacturer, is not guaranteed or endorsed by the publisher.

## Appendix

**Table:**

Interview questionnaire
Interview questionnaire for the purpose of thesis Kindly, could you respond the questionnaire in below for the study : Innovation Capabilities and Human Development Competitiveness in Education Sector: Evidence from UAE
**Part 1: Demographic data** **1. Age:** a. 18–25 b. 26–33 c. 34–41 d. 42–50 e. Over 51 **2. Gender:** a. Male b. Female **3. Education level:** a. Bachelors b. Master **4. Resident of UAE?** a. Yes b. No
Innovation capabilities:
**1.** How the innovation capabilities are contributing to achieve human development competitiveness in education? ? •
**2.** How the role of innovation capabilities in human development can play an important role to achieve competitiveness in education sector? •
**3.** Do you think the readiness of technological infrastructure in government sectors are assisting to improve the human development competitiveness in education sector? •
4. DO you think the UAE has integration innovation capabilities that enhancement to improve the human development competitiveness in education sector? •
**5.** How the UAE can apply the innovation capabilities to improve the human development competitiveness in education sector? •
**6.** Do you think the Innovation capabilities practical in education sector will enhance the UAE human development competitiveness?
**7.** Do you think the continuously measure of the human development KPIs in education is contributing to realize the points of weakness and strengths?
**8.** How the Innovation capabilities practical can contribute to improve the quality of education? •
**9.** Do you think the cooperation and integrated between government sectors will enhance the innovation capabilities to improve the human development competitiveness in education?
**10.** How the research and development can improve the quality of education in UAE and impact on human development competitiveness?
**11.** How the innovation strategy applied in education sector will improve the human development competitiveness in education and what the best practical can used? •
